# Diagnostic and therapeutic role of cholangioscopy in pancreaticoduodenal fistula

**DOI:** 10.1055/a-2814-5732

**Published:** 2026-03-11

**Authors:** Qian Zou, Long Xu, Lijuan Feng, Yun Qian, Lu Liu, Jingfeng Du

**Affiliations:** 1 558113 Department of Gastroenterology and Hepatology, Shenzhen University General Hospital, Shenzhen, China

We report a rare case of severe biliary pancreatitis complicated by a pancreaticoduodenal fistula. The patient was referred to our center after a failed endoscopic retrograde cholangiopancreatography (ERCP).

**Fig. 1:**
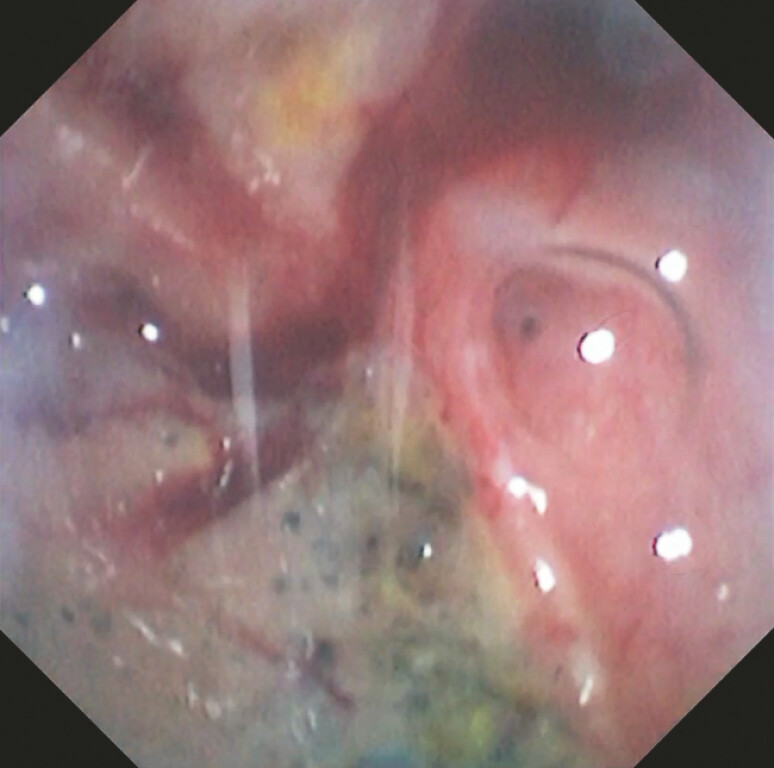
A cholangioscopic view of a pinpoint orifice with air bubbling inside the abscess cavity.

**Fig. 2:**
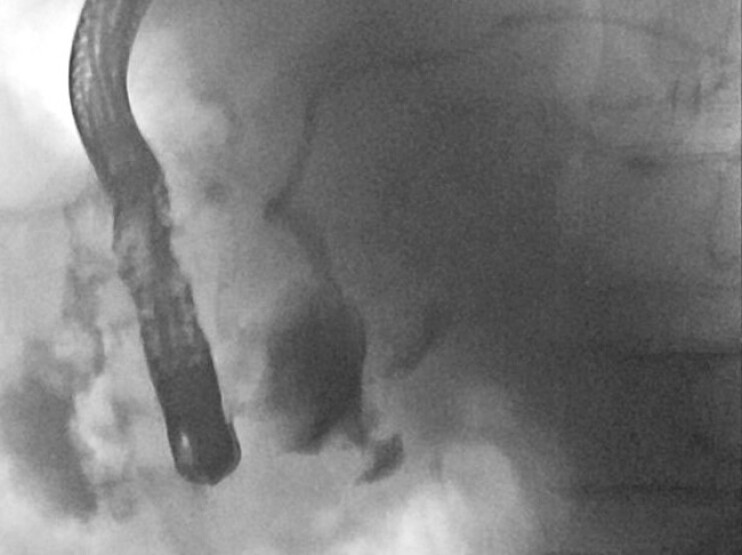
Contrast injection demonstrated direct communication between the abscess cavity and the main pancreatic duct.

During repeated ERCP, severe inflammatory edema of the descending duodenum completely obscured the major papilla. Careful inspection identified an abnormal orifice on the duodenal wall ( Video 1 ). A cholangioscope was advanced through this opening, revealing a large abscess cavity. Within the cavity, a pinpoint orifice with air bubbling was observed ( Fig. 1 ). Contrast injection demonstrated direct communication between the abscess cavity and the main pancreatic duct ( Fig. 2 ), confirming a pancreaticoduodenal fistula.

Diagnostic and therapeutic roles of cholangioscopy in pancreaticoduodenal fistulas.Video 1

**Fig. 3:**
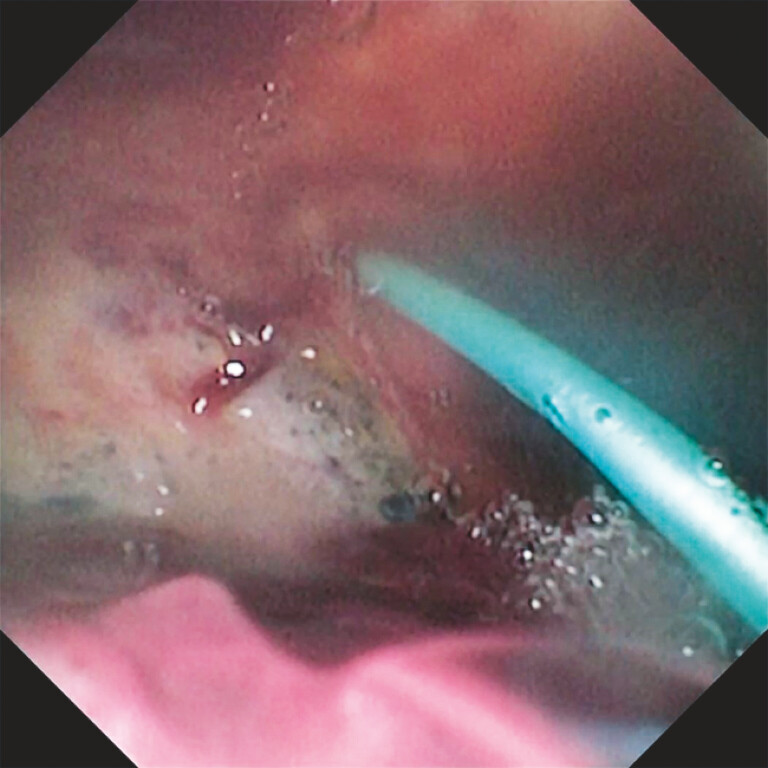
A pancreatic duct stent was placed across the fistulous tract to control pancreatic leakage.

**Fig. 4:**
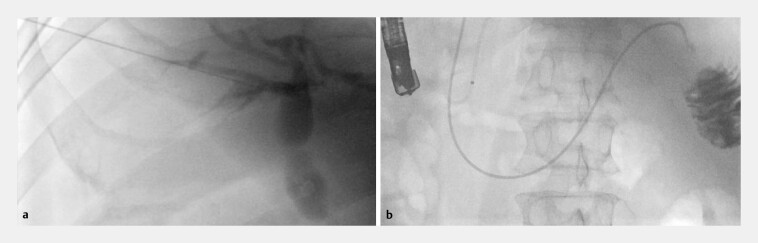
A combined PTCD–ERCP rendezvous procedure was performed. **a** Percutaneous transhepatic access was established. **b** A guidewire was advanced antegrade into the duodenum. PTCD–ERCP, percutaneous transhepatic cholangiodrainage–endoscopic retrograde cholangiopancreatography.

A pancreatic duct stent was placed across the fistulous tract to control pancreatic leakage ( Fig. 3 ). Biliary obstruction persisted because papillary access remained impossible. A combined percutaneous transhepatic cholangiography and drainage (PTCD)–ERCP rendezvous procedure was therefore performed ( Fig. 4 ), allowing the successful extraction of the obstructing common bile duct stone.

Subsequently, the pancreatic stent migrated into the abscess cavity and was retrieved under direct cholangioscopic visualization. A new pancreatic duct stent was inserted and anchored to the fistula wall using a titanium clip to prevent recurrent migration. Two additional plastic stents were placed to improve drainage.

**Fig. 5:**
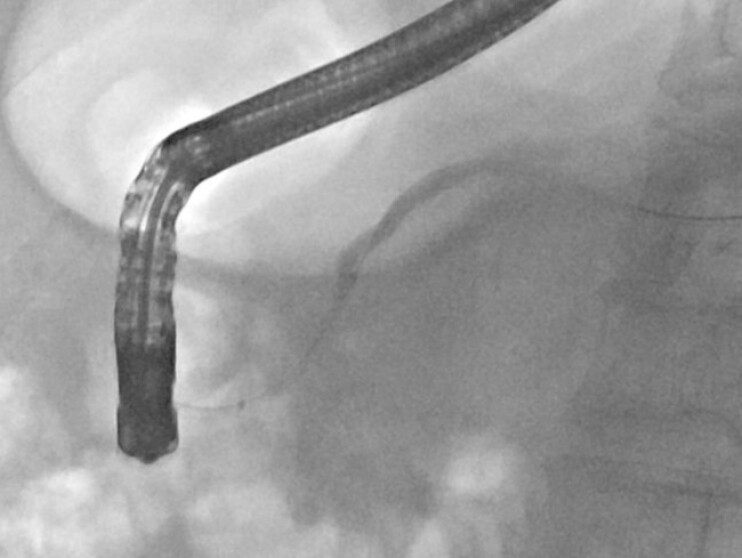
Pancreatography confirmed the complete healing of the pancreaticoduodenal fistula.

Four weeks later, the plastic stents were removed, and the fistula margins were approximated endoscopically around the indwelling pancreatic stent using through-the-scope twin clips combined with titanium clips. At a 6-month follow-up, near-complete fistula closure was observed, and the pancreatic stent was removed. Follow-up pancreatography confirmed complete closure ( Fig. 5 ).

This case highlights the pivotal diagnostic role of cholangioscopy in complex pancreaticobiliary fistulas and its therapeutic value in guiding endoscopic management. In addition, it demonstrates that the PTCD–ERCP rendezvous technique represents an effective salvage strategy in cases of an inaccessible papilla, offering a minimally invasive treatment option for complex pancreaticobiliary complications.

Endoscopy_UCTN_Code_TTT_1AR_2AI

